# Rare Image of Epidural Catheter Fracture in Lumbar Analgesia

**DOI:** 10.1155/2023/8880024

**Published:** 2023-08-28

**Authors:** Fiacro Jiménez-Ponce, Ylián Ramírez-Tapia, Erick Ariñez-Barahona, Jorge A. Nava-López, Sai Naveen Alla

**Affiliations:** ^1^Hospital Ángeles del Pedregal, Mexico City, Mexico; ^2^Hospital General de México “Dr. Eduardo Liceaga”, Mexico City, Mexico

## Abstract

**Objective:**

Accidental fracture of epidural analgesia catheters has a very low incidence of 2.5 per 100,000 anesthesia. A rare image of the fracture is reported.

**Methods:**

A 42-year-old female patient was attending a cesarean section eight years earlier to her consult. In the cesarean section, she received regional epidural anesthesia, and the main complaint was low back pain, specifically between the spinous processes L2 and L3. The somatic pain had been presenting intermittently for eight years. The sagittal section of magnetic resonance imaging of the lumbar spine showed a “golf club” image from the midline to the laminae of L2 and L3 with the subcutaneous tissue.

**Results:**

A small right hemilaminectomy was performed to remove the complete catheter, which did not adhere, but was coiled in the S-shape. The catheter was trapped between the left facets of L2 and L3 laterally than through the midline. Several risk factors and therapeutic procedures have been proposed.

**Conclusion:**

In a systematic review, 24 articles were reported on this specific issue. No surgical procedure and follow-up were informed by 8 authors. Surgical remotion by laminectomy was used in 9 articles, surgical explanation by skin incision was reported by 4 authors, and remotion by endoscopy was reported in 1 article. Two articles not reported solution.

## 1. Introduction

Complications derived from regional anesthetic procedures in the lumbar spine are rare. One of these complications is the accidental fracture of epidural analgesia catheters. This eventuality has been informed in regional anesthesia with a very low incidence of 2.5 per 100,000 anesthesia [1, 2]. The epidural regional anesthesia technique has been used since the beginning of the 20th century [1]. The material used to elaborate the catheters is diverse, including nylon, polyethylene, polyurethane, and polyamide. Since 2015, the manufacture of steel guides [2, 3] has begun.

This fracture is mainly due to the accidental excessive use of force when extracting the epidural tip. Visualization of catheters by a simple radiographic study has been reported, but this does not always occur. Extraction procedures have ranged from minimally invasive extraction to laminectomy approaches. The solution carries out within 24 hours to a maximum of years [4–8].

This article presents the case of a patient who underwent surgery with a diagnosis of lumbar granuloma with an inconclusive magnetic resonance image that was secondary to the presence of an epidural catheter accidentally left eight years ago.

## 2. Materials and Methods

### 2.1. Case Report

A 42-year-old female patient was attended by the neurosurgery service of Hospital Ángeles Pedregal in Mexico City. She had a significant history of hypothyroidism already treated with thyroid hormone and a cesarean section eight years earlier to her consult. In the cesarean section, she received regional epidural anesthesia. On this occasion, the main complaint was low back pain, specifically between the spinous processes L2 and L3. The somatic pain had been presenting intermittently for eight years. The frequency and intensity of this symptom were increasing. So she suffered almost daily for a few minutes and during exercise. In the beginning, the pain was evaluated with a Visual Analog Score (VAS) of two and resolved with nonsteroidal anti-inflammatory analgesics. There was a mild increase in volume in the lumbar region with painful sensation in the spinous processes L2 and L3. On general examination, neurologic exploration was normal.

**Figure 1:**
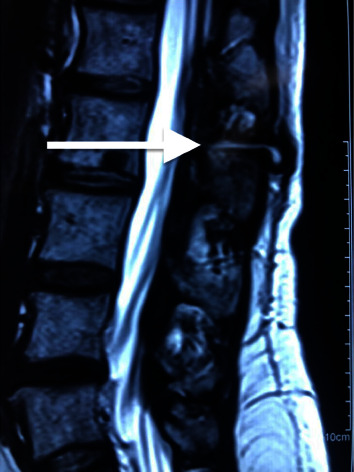
Sagittal magnetic resonance image of the lumbar spine in T2 sequence showing a hyperintense image like a “golf club” (arrow) from subcutaneous tissue between the spinous processes of L2-L3 through the interspinous ligament.

The catheter was not visualized by a simple X-ray image and did not show other alterations. The sagittal section of magnetic resonance imaging (MRI) of the lumbar spine showed a “golf club” image from the midline to the laminae of L2 and L3 to the subcutaneous tissue (Figure 1). In the axial projection, a similar image of a collection of approximately 25 mm in diameter was observed. These images were hypointense on T1 and hyperintense on T2. Any additional alterations were found within the spinal canal or intrathecal space. Either alteration was shown in the neuronal structures. A probable dermal sinus or granuloma of unknown etiology was a possible diagnosis. A surgical midline exploration or granuloma resection was suggested to the patient, but she refused it. The physicians ask her to keep a monthly follow-up and repeat MRI in six months. The patient came back one year after; the pain had been increased to a VAS rating of four and in frequency.

## 3. Results

**Figure 2:**
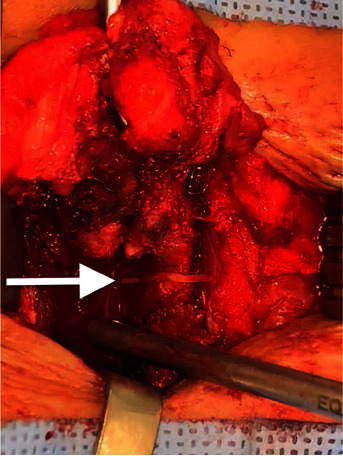
Approach to the lumbar region where the catheter (arrow) is partially observed between the processes of L2 and L3. It appears to be directed along the midline but is lateralized to the left of the facets.

**Figure 3:**
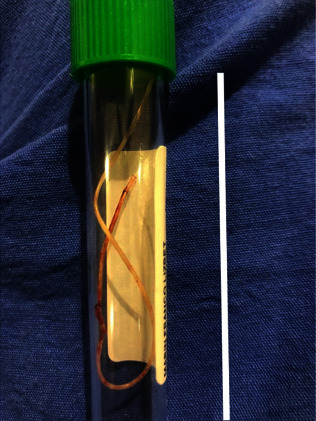
Photograph of the extracted catheter with an “S” shape and more than 12 cm in length. The bar in the figure has a length of 6 cm.

On this occasion, spine surgery was performed with a midline approach, gradually dissecting the lesion that was observed as gray-looking fibrosis from the subcutaneous region to the laminae of the vertebral body. One anesthetic perfusion catheter was found 10 mm outside of right recess (Figure 2). This catheter was continued into the epidural space for further 60 mm. A small right hemilaminectomy was performed to remove the complete catheter, which did not adhere, but was coiled in the S-shape (Figure 3). The catheter was trapped between the left facets of L2 and L3 laterally than through the midline.

Once the catheter was explanted, hemostasis was verified. Several samples for the culture were taken, and the surgical wound was closed by surgical planes. The patient was followed up three years later without complications or additional symptoms.

## 4. Discussion

In this review, we found 17 articles where this complication was reported (Table 1). Several risk factors and therapeutic procedures have been proposed. In addition, no surgical procedure and follow-up were informed by 8 authors. Surgical remotion by laminectomy was used in 9 articles, surgical explanation by skin incision was reported by 4 authors, and remotion by endoscopy was reported in one article. Two articles did not report solution. The median length of the catheter was 7.76 ± 5.45 cm. Local or neurological symptoms were reported in 8 articles.

In medicine, complications are always present in different spheres/stages. Regional anesthesia catheters can be broken accidentally during an anesthetic procedure. The reported cases range from 0.002 to 0.04% [24] or 0.000025% [1]. If the material is radiopaque, it facilitates localization in the immediate perioperative period, but in daily practice, their sections may be unnoticed and the material with which they were made may not be radiopaque.

It is considered that the fragmented catheter is inert and should not produce a reaction to a foreign body in the epidural space, but some studies reported inflammation after three weeks [1, 5].

Most of the reports that have been consulted report that the complication of catheter fracture resolves in hours or days [10, 11].

Different ways to solve the problem have also been recommended, and there is even a current study that advocates not to perform a maneuver in the first instance and that the patient shall only be medically supervised; depending on the clinical evolution, an intervention is performed [1, 3, 10, 13].

Complications have already been described and include pneumocephalus, abscesses, meningitis, neuropathy due to direct damage, dural tears, inadvertent administration of drugs intrathecally, arterial hypotension, ventilatory depression, and lack of sphincter control. Other more bizarre complications have been reported. For example, Tarukado reported broken catheter migration after four weeks [23].

The risk of rupture has been associated with degenerative changes in the spine that include foraminal stenosis, spondylolisthesis, hypertrophy of the ligamentum flavum, and a history of facetography [18]. The catheter can become trapped close to the ligamentum flavum, the posterior longitudinal ligament, the intervertebral foramen, pedicles, or the articular facets [18, 24].

To prevent this type of complication, it is suggested that the needle be checked so that it does not have imperfections at the tip and that the resistance of the catheter is adequate. In addition, it is recommended not to introduce the catheter more than 4 -5 cm into the epidural space to avoid rolling, twisting, or knotting [26]. The most vulnerable site for rupture is between 7 and 8 cm. This length is considered that when introducing more than 4 cm into the epidural space, there is a risk of knotting.

When a patient reports pain when withdrawing the catheter, nerve root avulsion may occur. So this catheter should be explanted under direct vision.

The symptoms most frequently associated with a catheter fracture are headache, local pain, and those that could be caused by nerve injury [18, 24].

Various procedures have been suggested in the literature [17, 26] to remove a catheter that is difficult to remove and thus prevent its rupture. For example:

- We place the patient in the same position that was punctured and wait from 15 to 30 minutes and then apply a slow and continuous extraction force, the force applied should be between 130 and 1000 gr to prevent it from breaking. Some authors report that the rupture can be produced from 2.6 kg.
- The patient can be subjected to sedation to facilitate muscle relaxation.
- Physiological saline solution be applied through the catheter trying to free the tube and eliminate probable twists.
- The Tuohy needle be applied parallel to the catheter, and then, we try to pull the catheter together with the needle.
- A CT scan of the spine be performed to find out the cause of the entrapment.
- It is suggested that the patient be subjected to general anesthesia to achieve muscle relaxation and position him in the same position in which the catheter was inserted.

From the surgical point of view, different surgical techniques have also been evaluated, including the removal of the foreign body by endoscopy and laminectomy. Regardless of the technique used to insert or remove the catheter, the patient's cooperation and catheter quality are factors that can influence rupture. If there are no symptoms, it is recommended not to remove the retained catheter, as this is not well documented, and the catheter can migrate and cause distant lesions.

Catheters of nylon or polyurethane 20 G are safer than teflon catheters 19 G because the last one has tendency to break during traction [2].

The other suggestion to avoid rupture of the catheter could be avoid getting approach parallel to or away from midline because this pathway increases the risk of rising lateral spine joints. Surgical sutures should not be used around the catheter. We should avoid introducing the catheter more than 5 cm into the peridural space. It is recommended to use nylon or reinforced polyurethane catheters. The catheter should not be removed if the puncture needle is still inserted because it increases the risk of rupture.

The present case only follows the case reported by Pinciroli with a catheter retained for 12 years, in which the catheter did not cause discomfort and was detected as it was radiopaque [22]. In this paper, the case presented a local inflammatory process that manifested itself eight years after the anesthetic procedure, a very characteristic and unusual image that can help other professionals to suspect the presence of this type of foreign body.

There are few publications about this anesthetic complication, but general information is common for location, time of diagnosis, and symptomatology. Posterior medical management includes several procedures. In Table 1, a compilation of papers is shown.

## 5. Conclusion

Fracture of the epidural catheter is an infrequent complication in regional anesthesia. Different brands have been associated with this side effect. A rare image in a golf club form is shown as chronically epidural catheter fractured, and the revision of the literature reported invasive and noninvasive managements.

## Figures and Tables

**Table 1:** A list of levels and symptoms involved in accidental fracture of epidural catheters and the ways of treating them.

Author	Year	Catheter	Level of the approach and fracture	Length of the catheter sectioned (cm)	Symptoms/sequelae	Treatment	Time to warm fracture

Tio et al. [9]	1979	Teflon	L2-L3	8	None	No surgery	Immediately
Moerman et al. [10]	1980	NA	L3-L4	NA	None	NA	Immediately
Crawford [11]	1985	Portex	NA	NA	None	No surgery	1 day
Staats et al. [12]	1995	Teflon	L3-L4	1	Lumbar stenosis, no sequelae	Laminectomy	Immediately
Collier [13]	2000	Portex	L2-L3 y L3-L4	4	Radicular compression syndrome	No surgery	Immediately
Nishio [14]	2001	Polyurethane catheter	L2-L3	5	None	Laminectomy	Immediately
Schummer and Schummer [15]	2002	Perifix	L3-L4	11	None	NA	Immediately
Dounas [16]	2002	Portex	L2-L3	6	None	Laminectomy	Immediately
Asai [17]	2001	Arrow	L3-L4	7.5	None	Laminectomy	Immediately
Castro-Rodríguez and López-Herranz [4]	2002	NA	L2-L3	0.9	Low back pain	No surgery	1 day
Ugboma [18]	2002	NA	L3-L4	9	None	Laminectomy	NA
Demiraran [19]	2006	Portex	L3-L4	4	Swelling lumbar region	Surgical remotion by skin incision	9 days
Drake [20]	2007	NA	NA	Tip sheared off	None	No surgery	NA
Eap [21]	2010	NA	L3-L4	NA	Low back pain	Surgical remotion by endoscopy	15 days
Mayorga-Buiza [1]	2012	NA	L2-L3	NA	None	No surgery	10 years
Abouhashem [5]	2013	NA	L3-L4	17	Back pain with pin prick sensation in spinal flexion	Surgical remotion by skin incision	Immediately
Mireles-Cano [6]	2014	Espinocat Plus	L2-L3 lateral foramen	5	None	Laminectomy	Immediately
Pinciroli and Fumagalli [22]	2015	Arrow-Teleflex	L3-L4	9	None	Surgical remotion by skin incision	12 years
Tarukado [23]	2015	Arrow-Teleflex	T11-T12	13	None	Laminectomy	7 weeks
Usar [3]	2015	Perifix® Soft Type 701	L3-L4	2	None	No surgery	9 months
Kim et al. [24]	2016	Racz	L5-S1 left facet	12	Severe radiating pain in the left leg	Laminectomy	1 year
Hippalgaonkar [25]	2017	Portex	L4-L5 into the muscle	19	None	Laminectomy	NA
Molina-García [7]	2017	NA	Tow case in L2-L3	14.0 and 13.0	None	Surgical remotion by skin incision	Immediately
Reena and Vikram [8]	2019	NA	L3-L4	1.5	None	No surgery	Immediately

Most frequent management is surgical remotion in perioperative time. However, in some cases, warming is late.

## Data Availability

This is a case report and review so the information is available in PubMed and Google Scholar.

## Abbreviations

VAS: Visual analog score
MRI: Magnetic resonance imaging
NA: Not available
L2: Lumbar 2
L3: Lumbar 3
L4: Lumbar 4
L5: Lumbar 5.
